# Aboriginal and Torres Strait Islander Peoples’ perceptions of foot and lower limb health: a systematic review

**DOI:** 10.1186/s13047-022-00557-0

**Published:** 2022-07-22

**Authors:** Sean Sadler, James Gerrard, Matthew West, Sean Lanting, James Charles, Angela Searle, Vivienne Chuter

**Affiliations:** 1 Discipline of Podiatry, School of Health Sciences, University of Newcastle, Newcastle, NSW Australia; 2 First Peoples Health Unit, Griffith University, Gold Coast, QLD Australia; 3 Discipline of Podiatry, School of Health Science, Western Sydney University, Campbelltown, NSW Australia

**Keywords:** Aboriginal and Torres Strait Islander Peoples, First Nation, Foot, Health status, Perceptions

## Abstract

**Background:**

Ongoing colonisation produces inequity in healthcare delivery and inequality in healthcare outcomes for Aboriginal and Torres Strait Islander Peoples. As a consequence, within the domain of lower limb health, foot disease has severe impacts for First Nations Peoples. Central to developing culturally safe healthcare and driving positive foot health change for First Nations Peoples, is the need for health professionals to develop understanding of First Nations perspectives of foot health. The aim of this systematic review was to evaluate studies investigating Aboriginal and Torres Strait Islander Peoples’ perceptions of foot and lower limb health.

**Methods:**

PubMeD, Ovid (Embase, Emcare, Medline), CINAHL, Informit Indigenous collection, and grey literature sources were searched to 23^rd^ July 2021. We included any published reports or studies that examined Aboriginal and Torres Strait Islander Peoples’ perceptions of foot and lower limb health, or meanings of, or attitudes to, foot and lower limb health.

**Results:**

Four studies with a total of 1515 participants were included. Studies found that Aboriginal and Torres Strait Islander people self-assessed foot health with a demonstrated ability to perceive their feet as healthy relative to Western clinical measures of peripheral blood supply and neurological function. Footwear, including ill-fitting or lack of footwear was considered a contributing factor to reduced foot and lower limb health. Foot pain affected up to 60% of participants with up to 70% of foot pain untreated. Lack of access to culturally safe health care delivered by culturally capable health professionals was perceived to contribute to worse foot and lower limb health outcomes.

**Conclusions:**

Aboriginal and Torres Strait Islander Peoples’ perceptions of foot and lower limb health are influenced by multiple complex interrelated factors. The limited number of studies in this area indicates ongoing failings to consult First Nations Peoples regarding their own lower limb and foot health. It is therefore essential that healthcare service and cultural capability implementation is led by Aboriginal and Torres Strait Islander Peoples in co-design. Urgent need for further research that exemplifies design and delivery of culturally safe care is required.

**Supplementary Information:**

The online version contains supplementary material available at 10.1186/s13047-022-00557-0.

## Background

This work includes the nomenclature; Indigenous, Aboriginal and Torres Strait Islander people, and First Nations Peoples. Neither singularly, nor collectively, do they adequately represent the immense diversity of language groups and cultures across this continent’s sovereign Traditional Custodians and rightful owners [1, 2].

## Introduction

Ongoing colonisation has been dismissive of First Nations health paradigms in place for millennia [3, 4]. This sees historical and current deficits in modern healthcare culminating in Aboriginal and Torres Strait Islander Peoples experiencing a burden of disease that is 2.3 times the rate of non-Indigenous Australians [5, 6]. A component of this burden relates to foot and lower limb health, with Aboriginal and Torres Strait Islander people having a 3–6 fold increased likelihood of experiencing a diabetes-related foot complication, and up to a 38 fold higher rate of amputation [7, 8]. Evidence of low rates of use of preventative foot care services by Aboriginal and Torres Strait Islander Peoples is likely to contribute to these outcomes [9–11] with such services being largely managed by entities [12] that have previously implemented policies including forcible removal of children [13] inflicting intergenerational trauma [14]. Effects of ongoing dispossession and distrust of healthcare systems linked to institutional and historical racism impacts the health and wellbeing of First Nations Peoples through manifestation of multifactorial disease such as diabetes [14–17].

The National Scheme’s Aboriginal and Torres Strait Islander Health and Cultural Safety Strategy 2020–2025 clearly articulates that cultural safety is determined by Aboriginal and Torres Strait Islander individuals, families, and Communities [18]. Provision of culturally safe diabetes-related services for Aboriginal and Torres Strait Islander Peoples have led to greater access to care, increased uptake of services, and enhanced patient outcomes [19–21].

Central to developing culturally safe frameworks across all levels of healthcare design and delivery is the inclusion of local Aboriginal and Torres Strait Islander voices [22]. Meaningful positive change can only be made to the poor foot health outcomes with a more complete understanding of First Nations perceptions and meanings of, or attitudes to good foot health. Working with and for First Nations Peoples, privileging their worldviews, taking anti-racist actions, engaging reciprocity, establishing trust, and establishing ongoing mutually beneficial relationships between research and Communities all underpin being able to facilitate a third space of combined knowledges that privileges First Nations perceptions in the care of their own lower limb and foot health [6, 18, 23, 24]. The experts in Aboriginal and Torres Strait Islander health are Aboriginal and Torres Strait Islander Peoples [3], therefore, the aim of this review was to systematically search the literature to identify Aboriginal and Torres Strait Islander Peoples’ perceptions of foot health.

## Methods

This systematic review was prospectively registered with PROSPERO (CRD42021277552) and has been reported in accordance with the PRISMA checklist [25]. An electronic database search of PubMeD (using Lit.search https://www.lowitja.org.au/litsearch from the Lowitja Institute), Ovid (Embase), Emcare, Medline, and CINAHL was conducted from database inception to July, 2021 (Additional file 1). Additional hand searches of grey literature sources were also conducted, including the Lowitja Institute, Menzies School of Health Research, and Australian Indigenous Health*Info*net Publications. Reference lists of included studies and review articles were also searched [26].

Inclusion criteria were any published reports or studies that examined Aboriginal and Torres Strait Islander Peoples’ perceptions of foot and lower limb health, or meanings of or attitudes to foot and lower limb health, including the presence or absence of conditions participants in the studies perceived as relating to foot health. No limits were placed on the method of foot health assessment. It was noted that, although eligible for inclusion, many patient-reported outcome measures (PROMs) have been developed with Western cultural perspectives and may not take account of Aboriginal and Torres Strait Islander perceptions of health, which are derived from their diverse cultures, value systems, and ways of knowing, being and doing, and therefore this was included in the study appraisal [27].

Two reviewers determined the electronic search terms and independently screened citations (AS and SS). Extraction of the study data was performed by one author (AS) and cross-checked by two authors (SS, JC). A descriptive synthesis of included study findings was performed. Methodological quality of the included studies was conducted by two authors (AS and SL) using two appraisal tools. Firstly, the Aboriginal and Torres Strait Islander Quality Appraisal Tool (QAT) was used to assess included studies through an Aboriginal and Torres Strait Islander lens [28]. This tool consists of 14 questions which are used to assess cultural safety of the study. Answer options include ‘yes’, ‘partially’, ‘no’, and ‘unclear’. Broadly, questions relate to Community engagement, First Nations leadership and governance, intellectual and cultural rights, and translation of findings to policy, practice, and Community. The quality appraisal outcomes for this tool were cross-checked by a First Nations researcher (MW). The second appraisal tool used was the Observational Study and Qualitative Study Appraisal Checklists designed by Health Evidence Bulletins – Wales [29]. This tool allows assessment of multiple observational study types (e.g. case–control, cohort, cross-sectional) and is a checklist rather than a scale. The appraisal tool focuses on four key domains: aims and outcomes of the study; population, bias, and follow-up; results, statistical analysis, and conclusions; and external validity. Disagreements were resolved between the two independent authors so arbitration by a third author was not required.

## Results

**Fig. 1:**
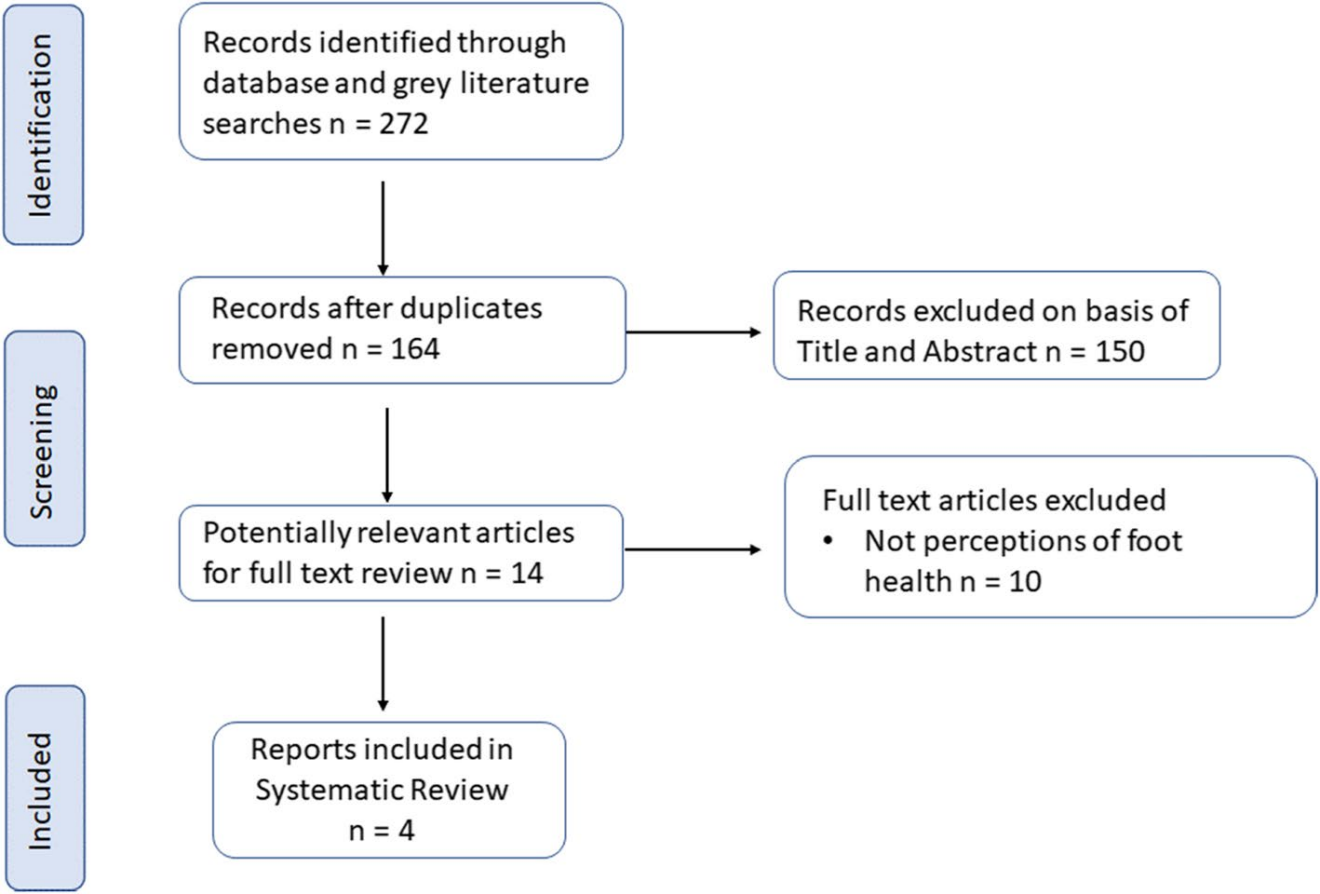
Flow diagram of systematic review inclusion and exclusion

Four studies involving 1515 participants met the inclusion criteria [5, 30–32] (Fig. 1). An overview of the included studies can be found in Table 1. Excluded studies are listed in Table 2. The methodological quality of the included studies is detailed in Additional files 2 and 3. With regard to the Aboriginal and Torres Strait Islander QAT, the questions with good outcomes were those regarding the use of appropriate Community consultation and protocols, Aboriginal and Torres Strait Islander research leadership, and research guided by an Indigenous paradigm with a strengths-based approach. In contrast, the studies had poor scores in regard to Aboriginal and Torres Strait Islander governance, access and ownership of intellectual and cultural property, and capacity strengthening for individuals. For the Health Evidence Bulletins quality assessment, all of the studies provided detailed information regarding study populations, aims, outcomes, and study method. The least favourably ranked questions were those concerning the relevance of the studies and the effects of confounding and bias. Relevant results in one population may not necessarily be applicable to other populations given the diverse populations (First Nation, Language Group, metropolitan, regional, rural and remote) of the included studies.

**Table 1:** Included studies

Author, location	Program type	Aims	Reported outcomes	Culturally responsive aspects
Charles 2018 [31] Metropolitan & regional NSW	Survey of injury *n* = 193 age range: 18 to 88 years mean age: 51 years	Determine effects of musculoskeletal injury on pain, mobility, weight gain, sleep, QOL and SEW through the use of a patient reported outcome measure, the AMIQ	60% of participants had an AMIQ Ankle summary score of 21–30 (moderate problem) 66% previous ankle injury 31% current ankle injury 22% rated current ankle injury as very or extremely painful 57% stated ankle injury contributed to weight gain 53% stated it contributed to sleep loss 72% rated it as a moderate or major problem in relation to sports or activity 70% had no treatment for the injury Sig. moderate to strong correlations between QOL, SEW and ankle injury	• AMIQ is culturally appropriate for Aboriginal Australians, by being patient centred, unambiguous, not time consuming, makes no assumptions, freely available, and at no cost • Original BFS questions that were deemed ambiguous or not appropriate for Aboriginal people were removed from the AMIQ (e.g., related to footwear) • The questions could easily be asked by a health professional, educator, or researcher, which is important due to literacy issues with some Aboriginal Community members
Jones 2001 [32] Metropolitan Adelaide or urban SA	Cross sectional study *n* = 1092 age (SD): 29.8 (17.2) years Focus groups *n* = 22 age range: 19 to 62 years	Gather data about issues of concern relating to foot health	63.1% experience foot pain, sig. associated with age# 10.9% constant WB pain 23.7% pain > 50% of WB 28.5% pain < 50% of WB 20.5% report foot problems: most common are ingrown toenails (with infection and pain) and Plantar fasciitis 70.7% wore shoes too small for their foot size Themes and quotes from focus groups are reported in the Results section	• Research was consistent with the guidelines on ethical matters in Aboriginal and Torres Strait Islander health research published by the NHMRC 1991 • Focus groups not taped at request of some attendees • Research conducted at neutral, nonthreatening venues controlled by Aboriginal Community organisations
West 2020 [30] Regional & rural NSW	Survey of participants attending culturally safe podiatry services *n* = 111 age (SD): 52.5 (16.3) years	Assessment of foot health measured through the use of a patient reported outcome measure, the FHSQ	FHSQ scores Pain: 75.7 ± 26.8 Function: 80.2 ± 25.2 Footwear: 53.9 ± 33.4 General foot health:62.0 ± 30.9 Diabetes sig. associated with lower levels of foot function#	• Podiatry clinics led by an Aboriginal Podiatrist and supported by an AHW • Clinics are designed to create an environment that is considerate of the spiritual, physical, social, and emotional world view of Aboriginal and Torres Strait Islander people • Includes an outreach program which occurs 3 to 4 times per year in a local Aboriginal Community facility, and operates as a drop-in clinic without any formal appointment times • AHW was present to assist participants if they had difficulty understanding the FHSQ questions
Wong 2005 [5] Torres Strait Islands and Northern Peninsula Area QLD	Focus groups and in-depth interviews in remote Communities *n* = 67 focus groups *n* = 30 individual interviews age: > 30 years	Qualitative study to achieve a better understanding of the perspectives and needs of Indigenous people with diabetes in the Torres Strait	Themes and quotes are reported in the Results section	• Research team consisted of Torres Strait Islander health workers who shared cultural background and dialect with the participants, and senior researchers in Indigenous and public health • Focus groups were held separately for men and women

*BFS* Bristol Foot Score, *AMIQ* Aboriginal Musculoskeletal Injury Questionnaire, *QOL* Quality of life, *SEW* Social and emotional well-being, *NHMRC* National Health and Medical Research Council, *SA* South Australia, *NSW* New South Wales, *QLD* Queensland, *FHSQ* Foot Health Status Questionnaire, *AHW* Aboriginal Health Worker,

^#^significant association *p* < 0.05, sig.: significantly, SD: standard deviation

**Table 2:** Excluded studies

Author	Name	Reason
Ballie et. al 2007	Improving organisational systems for diabetes care in Australian Indigenous Communities. BMC Health Services Research. 2007;7	Not Aboriginal and Torres Strait Islander perceptions of foot health
Ballestas et. al 2014	A metropolitan Aboriginal podiatry and diabetes outreach clinic to ameliorate foot-related complications in Aboriginal people. Australian & New Zealand Journal of Public Health. 2014;38(5):492–3	Not Aboriginal and Torres Strait Islander perceptions of foot health
Bandaranaike 2010	Stamping out diabetic foot in the Pilbara, Western Australia. Canberra: Services for Australian Rural and Remote Allied Health; 2010	Not Aboriginal and Torres Strait Islander perceptions of foot health
Charles 2017	The Aboriginal Multiple Injury Questionnaire (AMIQ): The development of a musculoskeletal injury questionnaire for an Australian Aboriginal population. Australian Indigenous HealthBulletin. 2017;17(3)	Not Aboriginal and Torres Strait Islander perceptions of foot health
Nannup et. al 2021	Keny Djena—‘first feet’: a story about Joobaitch (1830–1907) 2021	Not Aboriginal and Torres Strait Islander perceptions of foot health
Schoen et. al 2010	Health promotion resources for Aboriginal people: lessons learned from consultation and evaluation of diabetes foot care resources. Health Promotion Journal of Australia. 2010;21(1):64–9	Not Aboriginal and Torres Strait Islander perceptions of foot health
Warnock 2006	Mount Isa’s Indigenous Diabetic Foot Project. Deakin West, ACT: Services for Australian Rural and Remote Allied Health Inc; 2006	Not Aboriginal and Torres Strait Islander perceptions of foot health
Warnock 2006	An educational tool to assist with identification and management of the Indigenous diabetic foot. Deakin West, ACT: Services for Australian Rural and Remote Allied Health Inc; 2006	Not Aboriginal and Torres Strait Islander perceptions of foot health
Watson et. al 2001	Diabetic foot care: developing culturally appropriate educational tools for Aboriginal and Torres Strait Islander peoples in the Northern Territory, Australia. Australian Journal of Rural Health. 2001;9(3):121–6	Not Aboriginal and Torres Strait Islander perceptions of foot health
Whyatt et. al 2017	High Risk Foot: geographical inequities, importance of different diagnosis groups, forecast hospitalisations, and access to services. Perth, WA: Collaborative for Healthcare Analysis and Statistical Modelling (CHASM), School of Medicine, University of Western Australia; 2017	Not Aboriginal and Torres Strait Islander perceptions of foot health

West et al. (2020) found that Aboriginal people presenting to a culturally safe podiatry service had Foot Health Status Questionnaire (FHSQ) scores that were higher than a group of older non-Indigenous Australians also attending podiatry care [30]. The Aboriginal participant group had low levels of both clinically overt peripheral artery disease and peripheral neuropathy, with authors suggesting that this was likely to have contributed to a perception of healthy feet and higher FHSQ scores [30]. The study by Charles (2018) used the Aboriginal Multiple Injury Questionnaire (AMIQ) to examine ankle injury and any effects on quality of life, and social and emotional well-being [31]. This study revealed that Aboriginal and Torres Strait Islander Peoples’ perception of foot and lower limb health was poor with a high prevalence of ankle injury reported (31% current, 66% previous injury). This was associated with a significant level of pain (49% of participants reporting the very or extremely painful injuries) that affected walking, activity levels, and sleep. Despite these effects, 70% of people had no form of treatment for the injury, even though many of the participants lived in urban (Western Sydney) and large regional centres [31].

A mixed-methods study by Jones (2001) (*n* = 1092) also reported a high prevalence of foot pain (63.1%), with over one third of people reporting pain occurring > 50% of the time during standing and walking [32]. The focus groups (*n* = 22) provided further details of participants’ perceptions of foot and lower limb health (‘*almost everyone seems to have a foot problem of some sort’*), with pain and effects on activities of daily living (‘*unable to walk far because my feet hurt*’, ‘*As you get older and fatter, it’s hard to get down to your feet. It sounds so stupid, but having long toenails can stop you going out or walking around*’). Both extrinsic and intrinsic factors were identified by the focus group participants as contributing to their perceptions of poor foot health. Extrinsic factors included lack of information about commonly encountered foot problems such as callus and corns, and lack of availability of culturally safe foot care services, or services provided by a trained Aboriginal Health Worker (AHW), or Aboriginal Podiatrist. Another extrinsic factor highlighted by participants has impacting foot health was both bad shoes (*wrong style, size or material, or handed-down shoes*) and unaffordable shoes (‘*I was sent to the podiatrist at the hospital and they told me to go and buy a pair of shoes which cost over a hundred dollars. I can’t afford that, so I didn’t go back again to see them’*). Intrinsic factors included a sense of inevitability about the progression of lower limb complications *(‘I’ve got diabetes. My mother lost her leg and my aunty lost her leg. I know I will lose my legs eventually, so what's the point of worrying about it’*), a perception that it is normal for feet to hurt, and a lack of awareness that anything can be done, so most people just put up with the problems [32].

The study by Wong et al. (2005) used a qualitative approach to investigate the perceptions and needs of Indigenous people with diabetes in the Torres Strait, with the aim of promoting diabetes self-management strategies [5]. Although the primary aim of this study was not to assess foot health perceptions, a key theme that emerged from the analysis of focus groups data was ‘taking care of feet’. The participants’ quotes provide insight into their perceptions of what constitutes healthy feet. Factors mentioned by the participants included:

- Pulses

‘If there is no pulse, they may have to amputate.’ pp. 175

‘I usually get my granddaughter to check for my foot pulse, if she can’t feel it, or if she says that there is no pulse then I check it myself. I panic if she can’t feel it so then I check it myself.’ pp. 175

- Feeling or sensation

‘I check if I’ve got any feeling.’ pp. 175

- No Amputations

‘Yes, it is the most important thing, your legs, your feet. If you have half a leg you feel useless.’ pp. 175

‘I check my toes every time. Aunty … her toe it was numb and she had a stone in her toe.’ pp. 175

- No Infections, cuts or sores

‘If you get a sore then you must treat it straight away, don’t let it get infected.’ ‘When you have a shower make sure to check under your foot for cuts or anything like that.’ pp. 175

- Suitable footwear

‘I used to walk about barefoot, but now I wear shoes all the time, or sandal or thongs.’ pp. 175

‘Doctor said to wear shoes, covered footwear for bumps.’ pp. 175

- Regular personal and professional care

‘Check under your foot for cuts or anything like that, have your nails trimmed regularly.’ pp. 175

‘Rub moisturising cream on your foot to make sure there are no rashes or dry skin.’ pp. 175

‘Yes, any sore or cut you get, you should go straight to the Health Centre for dressing. It is very important.’ pp. 175

‘Well, I do it myself (cut toenails) because you (podiatrist) never come to see us. That’s why I lost my toe. You got to come and see us more often.’ pp. 175

Participants identified the importance of regular visits to health care professionals and the use of appropriate footwear as part of maintaining healthy feet. However, due to the remote geographical location of participants, there is intermittent access to health care professionals and limited access to good shoes which increased the risk of complications such as amputation.

## Discussion

The available literature identified evidence of high levels of perceived poor foot health and foot pain in Aboriginal and Torres Strait Islander populations. Personal experiences of family and Community with foot disease and related health outcomes (e.g., amputation) also shaped perceptions of good foot health and indicators of poor foot health. Participants describing a reliance on self-assessment reflected an understanding of the outcome but lack of access to culturally safe services to provide preventative care, rebutting negative stereotypes and demonstrating need for engagement, self-determination, and skillsets in culturally safe care provision. Similarly there was reference to inevitability of poor foot health which is consistent with fatalistic beliefs to overall health that have been documented in Aboriginal and Torres Strait Islander people [33] and are driven by: historical and continuing dispossession, socioeconomic inequality, lack of recognition of the importance of family, Community, and connection to Country in care provision, lack of provision of culturally safe care and care on Country, lack of social and emotional wellbeing and culture as an element of healing [34], and the persistent failure of Western models of health care delivery to improve First Nations Peoples’ health outcomes [33, 35, 36]. This is in stark contrast to more than 60 000 years of a successful First Nations Health paradigm prior to colonisation that supported long life expectancy and good health [37, 38].

A previous systematic review of foot health outcomes for First Nations Peoples demonstrated extreme disparities, with higher risk of developing foot disease and higher risk of catastrophic outcomes including infection, amputation and death compared to non-Indigenous Australians [8]. The limited understanding of Aboriginal and Torres Strait Islander People’s perceptions of foot health demonstrated by the dearth of studies available for inclusion in this systematic review is consistent with a long-standing reliance on a Western health care paradigm to unsuccessfully combat the severe impacts of foot disease on the health and wellbeing of First Nations Peoples in Australia [3, 4, 10, 39]. Previous systematic evaluation of foot care services for Aboriginal and Torres Strait Islander Peoples has demonstrated a fragmented approach to development and delivery of culturally safe foot care services, with lack of service evaluation and a lack of development of fundamental elements including workforce capability that are required to create more comprehensive availability of culturally safe care [40]. Consistent with these previous findings, the studies in this review demonstrated insufficient access to culturally safe care for Aboriginal and Torres Strait Islander people through both intermittent care provision and unsafe existing services. Participants identified that a lack of access to culturally safe health care contributed to worse foot and lower limb health outcomes.

Although the included studies involved diverse Aboriginal and Torres Strait Islander populations, one factor mentioned by all the studies as affecting foot health was footwear. This ranged from excluding questions relating to footwear, with the author of one study reporting that many Aboriginal people were not wearing closed-toe shoes but preferred slip-on open-toed footwear (e.g. due to hot weather) [31], that in remote areas no access to good shoes was a barrier preventing good foot care [5] and there being evidence of a majority of people (71% of *n* = 1092) wearing ill-fitting footwear [32], and reports of ‘good footwear’ being ‘unaffordable’. In the same study, focus group participants felt that ‘bad’ shoes were responsible for many foot problems [32]. Similarly the study investigating self-perceived foot health of Aboriginal and Torres Strait Islander People reported summary FHSQ scores that were higher than a population of older non-Indigenous Australians attending podiatry care [30]. However, the FHSQ footwear domain scores (50.7) of one of their groups were closer to the scores of populations with plantar heel pain (43.1 to 50.3, 44.4, 44.7) than healthy control groups (65.4, 68.8) [41–43]. These findings highlight the current role of footwear as a modifiable risk factor of poor foot health and the opportunity for provision of appropriate footwear to play a central role in improving foot health outcomes.

Cultural safety is born of Indigenous knowledges. The National Scheme’s Cultural Safety Strategy to eliminate racism from the healthcare system unites First Nations voices, the Aboriginal and Torres Strait Islander Health Curriculum Framework privileges First Nations voices, and the United Nations Declaration on the Rights of Indigenous Peoples states that Indigenous Peoples have the right to be free from any kind of discrimination, the right to self-determination, and the right to access all social and health services without any discrimination [44]. Therefore, based on the findings of this systematic review, particularly those relating to participants’ self-identified lack of access to culturally safe care as a contributing factor to their poor foot health, it is essential that future healthcare service delivery and cultural capability training of the podiatry workforce is led and co-designed by Aboriginal and Torres Strait Islander Peoples.

This review found a small number of studies and these included only Language Groups across First Nations in parts of the geographical areas now known as New South Wales, South Australia, and Queensland. Although there may be overarching themes relevant to a great number of First Nations Peoples, this work respects the diversity of First Nations Peoples and cultures and in no way wishes to promote homogenisation of an Indigenous population by promoting generalisability of results where only relevance to discrete populations may exist. The limited number of studies in this area indicates both ongoing failings to consult First Nations Peoples regarding their own lower limb and foot health, and an urgent need for further research. Such research needs to promote self-determination. Its methods should feature Participatory Action Research methods promoting social justice [45], as those without power have the right to feel safe and respected. Research should work with and for Aboriginal and Torres Strait Islander Peoples to investigate perceptions of foot and lower limb health across a greater range of diverse Language Groups and First Nations.

## Limitations

There are additional limitations of this review that need to be acknowledged. Importantly, many limitations stem from research being an unsafe term for First Nations Peoples impacted by ongoing colonisation [46]. Historically, research in Australia is based on Eurocentric worldview and featured unethical method and discriminatory design, that was devoid of consent and undertaken through an anthropological lens. It delivered invalid results and scarring negative stereotypes, and it justified colonisation of Australia with established power differentials [1, 6, 46, 47]. This has given First Nations Peoples good reason to avoid full engagement with research and researchers alike [6, 48, 49]. Because of past and continuing biases such as failing to report ethnicity in data [50] or recognising data sovereignty [51], research cannot be deemed by First Nations Peoples as completely safe to approach and use. Understandable mistrust is therefore a confounder in any research work, limiting the collection of rich data.

Regarding this study specifically, half of the articles included in this review were led by First Nations researchers and this will most likely have reduced influence of the aforementioned overarching limitation. Research though, even in co-design, is still not entitled to know everything about another culture, nor publish and/or disseminate all data and results. In any cross-cultural communication and work, humility must prevail, and respect be given to diverse cultural protocols and hierarchies. To infer competence in, or all knowledge of, another culture, no matter how much research and work has been undertaken, would be to reinforce ultimate power imbalance [52] and further make the research space unsafe.

A further limitation is that as large components of First Nations knowledges involve storytelling, yarning, dance, Country, ancestors, and spiritual aspects, many reported First Nations perceptions may be manipulated, distorted, or misrepresented through Western academy researching, reporting, editing, and publishing processes. Many PROMs have been developed with Western cultural perspectives, and this is the case for several studies in this review. Use of such tools may misrepresent First Nations perspectives. In this review process, Aboriginal researchers were involved in design of this review, and quality appraisal and data extraction (in addition to manuscript development); however, having them involved first-hand in the real-time search may have found additional studies for consideration and provided First Nations voice in moderating any disagreement for study inclusion/exclusion. Furthermore, although systematic searching included the Lowitja Institute, the Menzies School of Health Research, and Australian Indigenous Health*Info*Net Publications, which may limit the impacts of the academy’s processes, it should be noted too that First Nations perceptions of health may not be published in ‘searchable’ locations, and so omission of relevant data may also limit the study.

## Conclusion

Aboriginal and Torres Strait Islander Peoples’ perceptions of foot and lower limb health are influenced by multiple complex interrelated factors. These include the constructs of culture, family, kinship, Community, connection to Country, the cycle of life-death-life, ancestral and spiritual relationships, social and emotional wellbeing, and access to culturally safe healthcare. Urgent further research pursuing equity in healthcare delivery and equality in healthcare outcomes needs to deliver benefits for First Nations Peoples and involve more settings, health conditions, and severity of illness, to privilege the factors Aboriginal and Torres Strait Islander Peoples consider critical for optimal foot and lower limb health. Service provision and research must be co-designed and led by First Nations Peoples to ensure it is culturally safe.

## Supplementary Information

**Additional file 1.** PubMed searchstrategy as generated from the Lowitja Institute. (((((australia[mh] ORaustralia*[tiab]) AND (oceanic ancestry group[mh] OR aborigin*[tiab] ORindigenous[tw])) OR (torres strait* islander*[tiab])) AND medline[sb]) OR ((((au[ad]OR australia*[ad] OR australia*[tiab] OR northern territory[tiab] OR northernterritory[ad] OR tasmania[tiab] OR tasmania[ad] OR new south wales[tiab] OR newsouth wales[ad] OR victoria[tiab] OR victoria[ad] OR queensland[tiab] ORqueensland[ad]) AND (aborigin*[tiab] OR indigenous[tiab])) OR (torres strait*islander*[tiab])) NOT medline[sb]) AND English[la]) AND (foot or feet or lower‘limb’ or leg)

**Additional file 2.** Observational and Qualitative Study AppraisalChecklists, Health Evidence Bulletins – Wales.

**Additional file 3.** Aboriginal and Torres Strait Islander QualityAssessment Tool.

## Data Availability

Requests for further detail on the data collected in this study, or data sharing arrangements, can be submitted to Vivienne Chuter (Vivienne.chuter@newcastle.edu.au).

## Abbreviations

AHW: Aboriginal Health Worker
AMIQ: Aboriginal Musculoskeletal Injury Questionnaire
BFS: Bristol Foot Score
FHSQ: Foot Health Status Questionnaire
NHMRC: National Health and Medical Research Council
NSW: New South Wales
PROMs: Patient-reported outcomes measures
QAT: Quality appraisal tool
QLD: Queensland
SA: South Australia
SD: Standard deviation
SEW: Social and emotional well-being
QOL: Quality of life
