# Supplementary material for: Aboriginal and Torres Strait Islander Peoples’ perceptions of foot and lower limb health: a systematic review
Source: J Foot Ankle Res. 2022 Jul 22;15:55. doi: 10.1186/s13047-022-00557-0 (PMC9308327; doi:10.1186/s13047-022-00557-0)
Supplement: Supplementary file 1 — Additional file 1. PubMed searchstrategy as generated from the Lowitja Institute. (((((australia[mh] ORaustralia*[tiab]) AND (oceanic ancestry group[mh] OR aborigin*[tiab] ORindigenous[tw])) OR (torres strait* islander*[tiab])) AND medline[sb]) OR ((((au[ad]OR australia*[ad] OR australia*[tiab] OR northern territory[tiab] OR northernterritory[ad] OR tasmania[tiab] OR tasmania[ad] OR new south wales[tiab] OR newsouth wales[ad] OR victoria[tiab] OR victoria[ad] OR queensland[tiab] ORqueensland[ad]) AND (aborigin*[tiab] OR indigenous[tiab])) OR (torres strait*islander*[tiab])) NOT medline[sb]) AND English[la]) AND (foot or feet or lower‘limb’ or leg) [file 13047_2022_557_MOESM1_ESM.docx]

**Additional file 1:** PubMed search strategy as generated from the Lowitja Institute.

(((((australia[mh] OR australia*[tiab]) AND (oceanic ancestry group[mh] OR aborigin*[tiab] OR indigenous[tw])) OR (torres strait* islander*[tiab])) AND medline[sb]) OR ((((au[ad] OR australia*[ad] OR australia*[tiab] OR northern territory[tiab] OR northern territory[ad] OR tasmania[tiab] OR tasmania[ad] OR new south wales[tiab] OR new south wales[ad] OR victoria[tiab] OR victoria[ad] OR queensland[tiab] OR queensland[ad]) AND (aborigin*[tiab] OR indigenous[tiab])) OR (torres strait* islander*[tiab])) NOT medline[sb]) AND English[la]) AND (foot or feet or lower ‘limb’ or leg)
