# Supplementary material for: Aboriginal and Torres Strait Islander Peoples’ perceptions of foot and lower limb health: a systematic review
Source: J Foot Ankle Res. 2022 Jul 22;15:55. doi: 10.1186/s13047-022-00557-0 (PMC9308327; doi:10.1186/s13047-022-00557-0)
Supplement: Supplementary file 2 — Additional file 2. Observational and Qualitative Study AppraisalChecklists, Health Evidence Bulletins – Wales. [file 13047_2022_557_MOESM2_ESM.docx]

**Additional file 2: Observational and Qualitative Study Appraisal Checklists, Health Evidence Bulletins – Wales**

| **Articles** | Health Evidence Bulletins - Wales: Questions to assist with the critical appraisal of an observational study e.g. cohort, case-control, cross-sectional (Type IV evidence) and additional questions to assist with the critical appraisal of a qualitative study [29] | | **Charles[31]** | **Jones[32]** | **West[30]** | **Wong[5]** |
| --- | --- | --- | --- | --- | --- | --- |
| A.  What is this paper about? | 1. Is the study relevant to the needs of the project? (including Qualitative)* | | Y | Y | Y | Y |
|  | 2. Does the paper address a clearly focussed issue in terms of: | The population studied? | Y | Y | Y |  |
|  |  | (Case-control only) Is the case definition explicit and confirmed? | - | - | - |  |
|  |  | The outcomes considered? | Y | Y | Y |  |
|  |  | Are the aims of the investigation clearly stated? (including Qualitative)* | Y | Y | Y | Y |
| B.  Do I trust it? | 3. Is the choice of a cohort, case-control, cross-sectional study method appropriate? | | Y | Y | Y |  |
|  | OR (Qualitative only)*  3. Is the choice of a qualitative study method appropriate?   - What was this study exploring (eg behaviour/reasoning/beliefs)? - Do you think a quantitative approach could have equally/better addressed this issue? | |  |  |  | Y |
|  | 4. Is the population studied appropriate? | (Cohort study) Was an appropriate control group used – i.e. were the groups comparable on important confounding factors?  (Case-control study) Were the controls randomly selected from the same population as the cases? | N | Y | Y |  |
|  | OR (Qualitative only)*  4. Was the author's position clearly stated? | Has the researcher described his/her perspective?  Has the researcher examined his/her role, potential bias and influence? |  |  |  | Y |
|  | 5. Is confounding and bias considered? | Have all possible explanations of the effects been considered? | N | N | Y |  |
|  |  | (Cohort study) Were the assessors blind to the different groups? | - | - | - |  |
|  |  | (Cohort study) Could selective drop-out explain the effect? | N | N | N |  |
|  |  | (Case-control study) How comparable are the cases and controls with respect to confounding factors? | - | - | - |  |
|  |  | (Case-control study) Were interventions and other exposures assessed in the same way for cases and controls? | - | - | - |  |
|  |  | (Case-control study) Is it possible that overmatching has occurred in that cases and controls were matched on factors related to exposure? | - | - | - |  |
|  | OR (Qualitative only)*  5. Was the sampling strategy clearly described and  justified? | Check to see whether:  The method of sampling is stated or described  The investigators sampled the most useful or productive range of  individuals and settings relevant to their question  The characteristics of those included in the study are defined (and  are comparable to the wider population) |  |  |  | Y |
|  | 6. (Cohort study)  Was follow up for long enough | Could all likely effects have appeared in the time frame? | N | Y | Y |  |
|  |  | Could the effects be transitory? | - | - | - |  |
|  |  | Was follow up sufficiently complete? | Y | Y | Y |  |
|  |  | Was dose response shown? | - | - | - |  |
|  | OR (Qualitative only)*  6. Was an adequate description of the method of data  collection given? | Is the method of data collection described and justified?  How were the data collected (eg audiotape/videotape/field notes)?  If interviews were used, were the questions pre-tested?  If observation was used, is the context described and were  observations made in a variety of circumstances? |  |  |  | Y |
|  | OR (Qualitative only)*  7. Were the procedures for data analysis/interpretation  described and justified | Check to see whether:  A description is given of how the themes and concepts were identified in the data  The analysis was performed by more than one researcher  Negative/discrepant results were taken into account  The data were fed back to the participants for comment |  |  |  | N |
| C.  What did they find? | 7. Are tables/graphs labelled and understandable? | | Y | Y | Y |  |
|  | 8. Are you confident with the author’s choice and use of statistical methods, if employed? | | Y | Y | Y |  |
|  | OR (Qualitative only)*  8. What are the primary findings?  Consider whether the results: address the research question & are likely to be clinically important | |  |  |  | Y |
|  | 9. What are the results of this piece of research? Are the author’s conclusions adequately supported by information cited? | | Y | Y | Y |  |
|  | OR (Qualitative only)*  9. Are the results credible?  Were sequences from the original data presented (eg quotations) and were these fairly selected?  Is it possible to determine the source of the data presented (eg numbering of extracts)?  How much of the information collected is available for independent assessment?  Are the explanations for the results plausible and coherent?  Are the results of the study compared with those from other studies? | |  |  |  | Y |
| D.  Are the results relevant locally? | 10. Can the results be applied to the local situation? Consider differences between the local and study populations which could affect the relevance of the study (including Qualitative)* | | N | Y | Y | Y |
|  | 11. Were all important outcomes/results considered? (including Qualitative)* | | N | Y | Y | Y |
|  | 12. Is any cost information provided? | | N | N | N |  |
|  | 13. Accept for use ? (including Qualitative)* | | Y | Y | Y | Y |

Not all questions required for Qualitative studies. Questions required marked with Qualitative and *
