# Supplementary material for: Aboriginal and Torres Strait Islander Peoples’ perceptions of foot and lower limb health: a systematic review
Source: J Foot Ankle Res. 2022 Jul 22;15:55. doi: 10.1186/s13047-022-00557-0 (PMC9308327; doi:10.1186/s13047-022-00557-0)
Supplement: Supplementary file 3 — Additional file 3. Aboriginal and Torres Strait Islander QualityAssessment Tool. [file 13047_2022_557_MOESM3_ESM.docx]

**Additional file 3: Aboriginal and Torres Strait Islander Quality Assessment Tool**

|  | **Charles[31]** | **Jones[32]** | **West[30]** | **Wong[5]** | **Comments** |
| --- | --- | --- | --- | --- | --- |
| 1. Did the research respond to a need or priority determined by the community? | U | Y | U | Y |  |
| 2. Was community consultation and engagement appropriately inclusive? | Y | Y | Y | Y |  |
| 3. Did the research have Aboriginal and Torres Strait Islander research leadership? | Y | Y | Y | Y |  |
| 4. Did the research have Aboriginal and Torres Strait Islander governance? | U | U | U | U |  |
| 5. Were local community protocols respected and followed? | Y | Y | Y | Y |  |
| 6. Did the researchers negotiate agreements in regard to rights of access to Aboriginal and Torres Strait Islander peoples’ existing intellectual and cultural property? | U | U | U | U |  |
| 7. Did the researchers negotiate agreements to protect Aboriginal and Torres Strait Islander peoples' ownership of intellectual and cultural property created through the research? | U | Y | U | U |  |
| 8. Did Aboriginal and Torres Strait Islander peoples and communities have control over the collection and management of research materials? | P | P | P | P | Respectful and appropriate collection of data, but no control over data management |
| 9. Was the research guided by an Indigenous research paradigm? | Y | Y | Y | Y |  |
| 10. Does the research take a strengths-based approach, acknowledging and moving beyond practices that have harmed Aboriginal and Torres Strait peoples in the past? | Y | Y | Y | Y |  |
| 11. Did the researchers plan to and translate the findings into sustainable changes in policy and/or practice? | N | Y | Y | P |  |
| 12. Did the research benefit the participants and Aboriginal and Torres Strait Islander communities? | U | Y | Y | U |  |
| 13. Did the research demonstrate capacity strengthening for Aboriginal and Torres Strait Islander individuals? | Y | Y | Y | Y |  |
| 14. Did everyone involved in the research have opportunities to learn from each other? | U | Y | Y | Y |  |

Yes (Y), Partially (P), No (N), Unclear (U)
